# Changes in the characteristics of dental emergencies under the influence of SARS-CoV-2 pandemic: a retrospective study

**DOI:** 10.1186/s12903-021-01499-y

**Published:** 2021-04-03

**Authors:** Kan Wu, Chunjie Li, Zheng Yang, Shangchun Yang, Wenbing Yang, Chengge Hua

**Affiliations:** 1 State Key Laboratory of Oral Diseases, National Clinical Research Center for Oral Diseases, Department of Medical Affairs, West China Hospital of Stomatology, Sichuan University, Chengdu, China; 2 State Key Laboratory of Oral Diseases, National Clinical Research Center for Oral Diseases, Department of Head and Neck Oncology, West China Hospital of Stomatology, Sichuan University, Chengdu, China; 3 State Key Laboratory of Oral Diseases, National Clinical Research Center for Oral Diseases, Department of Dental Emergency and General Dentistry, West China Hospital of Stomatology, Sichuan University, Chengdu, China

**Keywords:** Dental emergency patients, Demographics, Diagnoses, Treatment, SARS-COV-2

## Abstract

**Background:**

Further understanding of the distribution and changing characteristics of dental diseases is of great significance for all dental emergency centers for strengthening the medical staff’s treatment knowledge abilities and effective use of emergency resources in the face of public health emergencies involving highly infectious respiratory diseases.

**Methods:**

The medical records of 4158 dental emergency patients in 2019 and 2020 were retrospectively analyzed and divided into pre-SARS-COV-2 group and SARS-COV-2 group according to time. The demographic data, date and time, diagnosis, and treatment methods of the two groups were statistically described, and the chi-squared test was used to analyze the differences. The medical records of 4158 dental emergency patients during the same period of two years in 2019 and 2020 were retrospectively analyzed and divided into SARS-COV-2 pre-group and SARS-COV-2 group according to time. The demographic data, date and time, diagnosis and treatment methods of the two groups were statistically described, and the chi-square test was used to determine the differences.

**Results:**

During the SARS-COV-2 pandemic, the number of dental emergency visits increased by 29.7%. During the pandemic, males (n = 286, 58.1%) were more likely to visit dental emergency centers for trauma than females (n = 206, 41.9%) (*P* < 0.05); females (n = 242, 60.8%) were more likely to visit dental emergency centers for acute gingivitis and acute pericoronitis than males (n = 156, 39.2%) (*P* < 0.05). A major change in diagnosis was related to acute pulpitis (K04.0) and acute apical periodontitis (K04.4), which increased by 9.2%; acute gingivitis (K05.0) and acute pericoronitis (K05.2) increased by 3.5%; open wound of the lip and oral cavity (S01.5) decreased by 17.9%; other conditions (non-emergency diseases) increased by 6.8%, compared with the pre-SARS-COV-2 period. Among the treatment modalities, during the pre-SARS-COV-2 period, 304 patients (17.7%) received a prescription for antibiotics and analgesics, and 1485 (86.5%) received a prescription for local treatment. During the SARS-COV-2 period, 958 (39.2%) received a prescription for antibiotics and analgesics, and 1636 (67.0%) received a prescription for local treatment.

**Conclusion:**

SARS-COV-2 pandemic led to changes in the characteristics of dental emergency patients. Trauma, acute pulpitis, and acute periodontitis are the leading reasons patients refer to dental emergency centers. Dental emergency centers should optimize treatment procedures, optimize the staff, and reasonably allocate materials according to the changes to improve the on-site treatment capacity and provide adequate dental emergency care.

## Background

Dental emergency patients often need emergency treatment because of acute facial or dental pain, bleeding, trauma, and other conditions [1]. Dental emergencies progress quickly because of the acute and complex nature of some conditions in patients. Cases that do not progress acutely should be triaged, such as the fracture of a prosthetic device (e.g., a denture), because they do not progress acutely. The composition and epidemiological characteristics of dental diseases in emergency centers vary from region to region [1, 2]. Previous studies have summarized the characteristics of visits to emergency dental centers. For example, concerning time distribution, the number of visits to emergency dental centers at weekends is more than that during the week [3], and visits to emergency dental centers peak at night [3, 4]. Gender and age exhibit different features in all kinds of emergency visits [3–5], and the main reasons for visits are pulpitis, trauma, and bleeding [5].

The composition and characteristics of emergency dental care are influenced by environmental changes, lifestyle, and economic and sociocultural factors [6]. With the spread of SARS-COV-2 worldwide, people adopted home isolation to reduce going out and gatherings, resulting in changes in the population’s living environment, psychological status, and lifestyle [7]. The composition of dental emergency diseases also exhibits different changes and characteristics. Most of the risk factors associated with dental emergencies are considered preventable [6, 8]. Characteristics of dental emergency patients diagnosed with SARS-COV-2 have been reported. There is a lack of comparison with the situation before the SARS-COV-2 pandemic [9] to analyze changes in emergency diseases’ classification [1, 9].

Hospitals and clinics try to reduce or stop visits to ensure doctors’ and patients’ safety under the impact of the SARS-COV-2 pandemic. At the same time, the dental treatment process has changed accordingly [1, 9, 10], and dental protection needs to be strengthened during treatment, reduce the use of some dental appliances that cause spatter, change treatment options and drug use, and improve dental emergency care [10, 11]. Therefore, knowledge about the dental emergency diseases’ classification and changes in the demographic characteristics of the dental emergency center in the face of intense public health emergencies of respiratory infectious disease can improve the efficiency of treatment of dental emergency patients and provide dental emergency personnel, supplies, and emergency technical expertise to supply important reference data [12].

In this study, we collected the demographic data, day and time, diagnosis, treatment modality data of patients visiting the dental emergency center during the SARS-COV-2 pandemic from January 20 to March 8, 2020, through retrospective analysis. Simultaneously, patients in the emergency center in the same period (i.e., 2019) were selected as the control group to compare and analyze the demographic characteristics and disease composition of emergency dental patients and the treatment modalities before and changes during the SARS-COV-2 pandemic. The present study aimed to explore the characteristics of dental emergencies, provide references for the diagnosis and treatment of critical illnesses in dental emergency centers, and provide a scientific basis for the organization and management of equipment, materials, technology, and personnel in dental emergency centers.

## Methods

### Data sources and grouping

All the patients presenting to the dental emergency service of the National Clinical Research Center for Oral Diseases, Department of Dental Emergency, West China Hospital of Stomatology, Sichuan University, located in Chengdu, Sichuan, China, were included in the present study. It is an important dental emergency center in western China. The emergency center is open from Monday to Sunday from 0:00 to 24:00 every day. Information, including dental emergency patients’ demographic data, diagnoses, treatment methods, and the use of antibiotics and analgesics collected from January 20 to March 8, 2020, comprised the SARS-COV-2 group data.

In order to better compare the changes of dental emergency patient visits before and after the epidemic, and avoid the influence of time, season and other mixed factors, we set the data during the same period (from January 21 to March 10, 2019) as the control group (pre-SARS-COV–2 group). Screening criteria included data from 4158 visitors with definitive disease diagnosis and complete medical records in the dental emergency center before the pandemic.

### Classification

A retrospective investigation of the patients was carried out to analyze demographic data: sex (male, female), age (0–18 years old in the juvenile group, 19–45 years old in the youth group, 46–65 years old in the middle-aged group, ≥ 66 years old in the elderly group), the period (weekly trend changes, daily trend changes), the dental emergency treatment approaches, including drug use (antibiotics/analgesics) and local treatment.

### Diagnoses

According to the standards of the International Classification of Diseases, 10th edition (ICD-10) [13, 14], the following seven categories of dental emergencies preliminarily diagnosed by pre-hospital physicians were included in this study:

- Group 1: Acute pulpitis (K04.0) and/or acute apical periodontitis (K04.4)
- Group 2: Acute gingivitis (K05.0) and/or acute pericoronitis (K05.2)
- Group 3: Temporomandibular joint disorders (K07.6)
- Group 4: Cellulitis and abscess of the oral cavity (K12.2)
- Group 5: Open wound of the lip and oral cavity (S01.5)
- Group 6: Fracture of tooth (S02.5)
- Group 7: Others (non-emergency diseases, including diagnoses related to a prosthesis, aesthetic, recall, or maintenance)’

### Statistical methods

Statistical analyses were performed using SPSS (version 20.0). The data were normally distributed and presented as means ± standard deviations. A chi-squared test was used to analyze the distribution between groups. Statistical significance was defined at P < 0.05.

## Results

### Demographic data

**Fig. 1:**
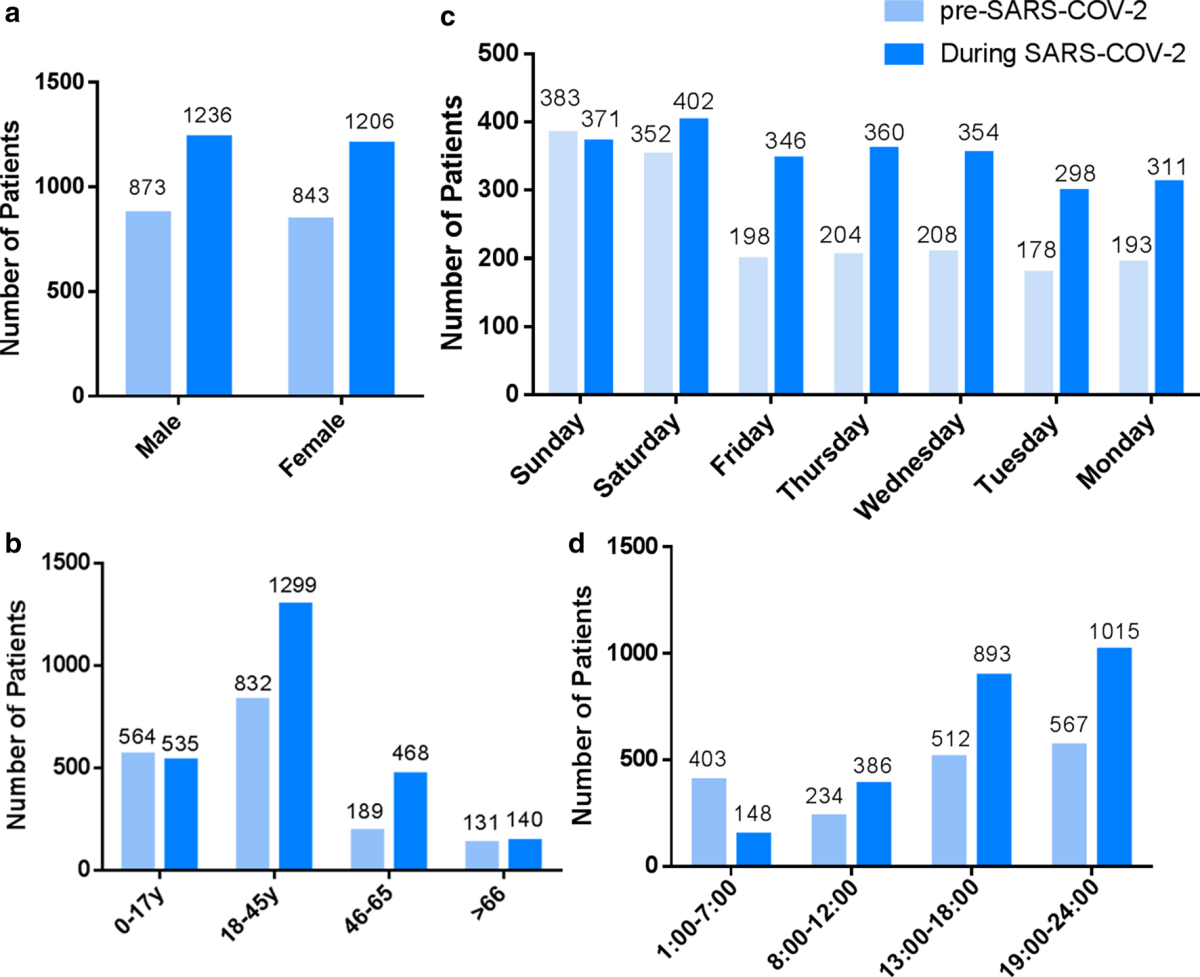
Distribution of patients by sex, age, day, and time

A total of 4158 emergency patients were included in the study, consisting of 1716 and 2442 patients in the pre-SARS-COV-2 and SARS-COV-2 groups, respectively. There were males (n = 873, 50.9%) and females (n = 843, 49.1%) in the pre-SARS-COV-2 group, with a male-to-female ratio of 1.04:1. Concerning age composition, 564 (32.9%) patients were 0–17 years of age in the juvenile group, 832 (48.5%) were 18–45 in the youth group, 189 (11.0%) were 46–65 in the middle-aged group, and 131 (7.6%) were ≥ 65 in the elderly group. The mean age was 24.7 ± 16.7 years. In the SARS-COV-2 group, there were males (n = 1236, 50.6%) and females (n = 1206, 49.4%), with a male-to-female ratio of 1.02:1. Concerning age groups, 535 (21.9%) were 0–17 years old in the juvenile group, 1299 (53.2%) were 18–45 in the youth group, 468 (19.2%) were 46–65 in the middle-aged group, and 140 (5.7%) were ≥ 65 in the elderly group. The mean age was 33.0 ± 19.4 years (Fig. 1a, b).

### Distribution of day and time

The number of dental emergency patients in the SARS-COV-2 period increased by 29.73% compared with the number of patients admitted to the emergency center before the SARS-COV-2 pandemic, with the average daily emergency visits increasing from 35.0 to 49.8. Concerning the day distribution, the highest number of dental emergency cases in the pre-outbreak period was on Sundays (n = 383, 22.3%). The minimum day was Tuesdays (n = 178, 10.4%); the highest number of dental emergency cases during the outbreak was on Saturdays (n = 402, 16.5%), and the lowest was Tuesdays (n = 298, 12.2%). Compared with the SARS-COV-2 period, the patients were more numerous in the weekend visits. During the visit period, both groups of patients in the emergency dental care had a visit peak from 19:00 to 24:00, with 567 patients (33.0%) in the pre-SARS-COV-2 group and 1015 patients (41.6%) in the SARS-COV-2 period group (Fig. 1c, d).

### Distribution of different emergency conditions

Table 1 shows the changing characteristics of disease entities. The main reasons for emergency visits were acute pulpitis (K04.0) and acute apical periodontitis (K04.4) (n = 858, 35.1%), Acute gingivitis (K05.0) and acute pericoronitis (K05.2) (n = 529, 21.7%), open wounds of the lip and oral cavity (S01.5) (n = 492, 20.1%) in the SARS-COV-2 group. The main reasons for emergency visits were open wounds of the lip and oral cavity (S01.5) (n = 695, 38.0%), acute pulpitis (K04.0), acute apical periodontitis (K04.4) (n = 413, 25.9%), acute gingivitis (K05.0), and acute pericoronitis (K05.2) (n = 314, 18.2%) in the pre-SARS-COV-2 group. The difference was statistically significant (*P* < 0.05). The other significant changes were in other non-emergency conditions in the SARS-COV-2 group (n = 257, 10.5%) compared with those in the pre-SARS-COV-2 group (n = 51, 3.7%) (*P* < 0.05) (Table 1).

**Table1:** Diagnosis in relation to demographic characteristics

Diagnosis	Pre-SARS-COV-2 group					During-SARS-COV-2 group					*P* value
	Patients n (%)	Mean age	Gender			Patients	Mean age	Gender			
			Male n (%)	Female n (%)	*P* value			Male n (%)	Female n(5)	*P* value	
Group1	413 (25.9%)	31.4 ± 14.8	206 (49.9%)	207 (50.1%)	0.642	858 (35.1%)	38.4 ± 17.9	423 (49.3%)	435 (50.7%)	0.339	0.000
Group2	314 (18.2%)	30.9 ± 12.2	121 (38.5%)	193 (61.5%)	0.000	529 (21.7%)	32.8 ± 14.7	156 (39.2%)	242 (60.8%)	0.000	0.005
Group3	24 (1.4%)	31.8 ± 13.9	10 (41.7%)	14 (58.3%)	0.364	56 (2.3%)	38.1 ± 26.0	33 (58.9%)	23 (41.1%)	0.008	0.043
Group4	161 (9.6%)	35.5 ± 15.1	80 (49.7%)	81 (50.3%)	0.752	227 (9.3%)	40.7 ± 19.4	122 (53.7%)	105 (46.3%)	0.322	0.715
Group5	695 (38.0%)	15.2 ± 15.9	405 (58.3%)	290 (41.7%)	0.000	492 (20.1%)	19.8 ± 17.9	286 (58.1%)	206 (41.9%)	0.000	0.000
Group6	58 (3.2%)	23.5 ± 12.1	31 (53.4%)	27 (46.6%)	0.729	23 (0.9%)	32.2 ± 17.6	10 (43.5%)	13 (56.5%)	0.492	0.000
Group7	51 (3.7%)	26.5 ± 12.3	20 (39.2%)	31 (60.8%)	0.091	257 (10.5%)	33.5 ± 21.0	127 (49.4%)	130 (50.6%)	0.685	

Group1: Acute Pulpitis (K04.0) and/or Acute apical periodontitis (K04.4)

Group2: Acute gingivitis (K05.0) and/or Acute pericoronitis (K05.2)

Group3: Temporomandibular joint disorders (K07.6)

Group4: Cellulitis and abscess of mouth (K12.2)

Group5: Open wound of lip and/or oral cavity (S01.5)

Group6: Fracture of tooth (S02.5)

Group7: Others (non-emergency diseases, including diagnoses related to prosthesis, aesthetic, recall or maintenance)

Moreover, during the SARS-COV-2 pandemic, females (n = 242, 60.8%) were more likely to visit the emergency center due to acute gingivitis and acute pericoronitis than males (n = 156, 39.2%), while males (n = 286, 58.1%) were more likely to visit the emergency center due to open wounds of the lip and oral cavity than females (n = 206, 41.9%). The low age group (19.8 ± 17.9) was prone to open wounds of the lip and oral cavity, while the middle-aged group (40.7 ± 19.4) was more prone to visit because of cellulitis and abscess of the oral cavity (Table 1).

### Treatment and the use of drugs

In the pre-SARS-COV-2 group, of 1716 patients visited in the dental emergency service, 304 (17.7%) received a prescription for antibiotics and analgesics; 1485 (86.5%) received a prescription for local treatment. The diseases most commonly treated with antibiotics and analgesics are cellulitis and abscesses of the oral cavity (n = 76, 47.2%), other (n = 12, 23.5%), acute gingivitis, and acute pericoronitis (n = 61, 19.4%). The conditions most commonly treated with local treatment are open wounds of the lip and oral cavity (n = 655, 94.2%), acute pulpitis, acute apical periodontitis (n = 364, 88.1%), acute gingivitis, and acute pericoronitis (n = 259, 82.5%). In the SARS-COV-2 group, of 2442 patients visited in the dental emergency service, 958 (39.2%) received a prescription for antibiotics and analgesics, and 1636 (67.0%) received a prescription for local treatment. The conditions most commonly treated with antibiotics and analgesics are acute gingivitis and acute pericoronitis (n = 227, 44.9%), cellulitis and abscesses of the oral cavity (n = 99, 43.6%), acute pulpitis and acute apical periodontitis (n = 208, 42.3%). The diseases most commonly treated with local treatment are open wounds of the lip and oral cavity (n = 474, 96.3%), acute gingivitis and acuteæ pericoronitis (n = 367, 69.4%), cellulitis and abscesses of the oral cavity (n = 156, 68.7%) (Table 2).

**Table 2:** Use of Antibiotics and/or Analgesics and treatment by diagnosis

Diagnosis	Patients n	Pre-SARS-COV-2		Patients n	During-SARS-COV-2	
		Antibiotics/Analgesics n(%)	local treatment n(%)		Antibiotics/Analgesics n (%)	Local treatment n (%)
Group1	413	45 (10.9%)	364 (88.1%)	858	219 (25.5%)	583 (67.9%)
Group2	314	61 (19.4%)	259 (82.5%)	529	227 (44.9%)	367 (69.4%)
Group3	24	1 (4.2%)	7 (29.2%)	56	2 (3.6%)	10 (17.9%)
Group4	161	76 (47.2%)	132 (82.0%)	227	99 (43.6%)	156 (68.7%)
Group5	695	103 (14.8%)	655 (94.2%)	492	208 (42.3%)	474 (96.3%)
Group6	58	6 (6.1%)	31 (53.4%)	23	6 (26.1%)	7 (30.4%)
Group7	51	12 (23.5%)	37 (72.5%)	257	197 (76.7%)	39 (15.2%)

Group1: Acute Pulpitis (K04.0) and/or Acute apical periodontitis (K04.4)

Group2: Acute gingivitis (K05.0) and/or Acute pericoronitis (K05.2)

Group3: Temporomandibular joint disorders (K07.6)

Group4: Cellulitis and abscess of mouth (K12.2)

Group5: Open wound of lip and/or oral cavity (S01.5)

Group6: Fracture of tooth (S02.5)

Group7: Others (non-emergency diseases, including diagnoses related to prosthesis, aesthetic, recall or maintenance)

## Discussion

During the SARS-COV-2 pandemic, the number of emergency dental patients increased by 29.7%, and the average daily visits increased by 14.8%, mainly due to the high risk of dental treatment under the influence of the SARS-COV-2 pandemic, which led to the decrease or closure of the general outpatient clinics of stomatological hospitals or clinics in China. This decreased regular examinations and treatment of patients on time, aggravating the condition and patient pain and suffering, which increased the demand for dental emergency services and the number of emergency visits [7, 15]. Regarding the weekly variations in the number of visit days, the number of patients visited at the weekend in the previous epidemic period was higher than the number of patients visited during the week, consistent with the distribution of dental emergency time and visits in South Korea [16]. With the outbreak, this difference became less pronounced, and the distribution of daily and weekly visits after the outbreak decreased, which might be related to increased demands for emergency dental services. Concerning the visit time, the pre-SARS-COV-2 group in this study and another study on emergency visits in South Korea [16, 17] showed a similar distribution of visit times of patients in a dental emergency, both of which had a peak visit time at night. During the epidemic, the number of patients visiting during the day increased except for the night peak [2, 18], which was related to decreased dental outpatient service during the SARS-COV-2 pandemic and patients’ demands for medical emergency treatments. Therefore, dental emergency centers in a region should increase their personnel and material support in the face of respiratory infectious disease emergencies to cope with the increased emergency needs of dental emergency patients due to the closure of clinics [11].

Concerning gender, our data showed a relatively balanced distribution of emergency visits between males and females. In contrast, other studies found that men are more likely to visit emergency centers [19]. The present study found that males are more likely to have trauma visits than females, while females are more likely to have acute gingivitis and acute periodontal disease visits than males. Such differences might be attributed to males’ and females’ different social function roles; therefore, males are more likely to engage in dangerous occupations [19, 20]. Studies have shown that women pay more attention to oral healthcare than men [19, 21]. Therefore, when gingivitis and periodontitis occur, females are more likely to seek medical treatment. Meanwhile, females feel less uncomfortable with pain than males, which might also increase emergency visits [22].

By comparing the disease composition of oral emergency patients before and during the SARS-COV-2 pandemic, acute pulpitis (K04.0), acute apical periodontitis (K04.4), acute gingivitis (K05.0), acute pericoronitis (K05.2), open wound of the lip and oral cavity (S01.5) comprised > 70% of the main types of dental emergency conditions, consistent with Jingjing qiu and Jingjing’s results against the Characteristics of Endodontic Emergencies during SARS-COV-2 pandemic [21]. The main complaint was toothache, with its own characteristic nature of the pain, location, and duration. During the SARS-COV-2 pandemic, the admission of patients with dental and trauma to the dental emergency center declined, but acute pulpitis (K04.0) and acute apical periodontitis (K04.4) significantly increased. It is possible that during the pandemic, home quarantine and preventive measures which forced people to stay at home more, thereby reducing the risk and occurrence of injuries [19, 22, 23]. The increase in the proportion of acute pulpitis has been related to perhaps lack of attendance to the general dentists during the SARS-COV-2 pandemic. In addition, there was a significant increase in the proportion of non-emergency cases during the pandemic, such as prosthesis, aesthetic, recall, or maintenance, which resulted in rapid disease progression and unbearable pain in patients due to their inability to return to their scheduled treatments, ultimately seeking emergency treatment. This might be due to the closure of dental medical facilities during the pandemic, with patients unable to maintain their scheduled follow-up treatments [24].

Due to the high risk of SARS-COV-2 transmission via dental emergency treatments, therapeutic instruments and equipment have been limited, such as high-speed turbines used in endodontic treatment and ultrasonic scalers in periodontics. The selection of emergency treatment programs was also affected due to changes during the pandemic. Therapeutic instruments and equipment were mainly for relieving acute symptoms because the diagnosis and treatment principles during the SARS-COV-2 pandemic necessitate relief of pain, elimination of inflammation, hemostasis, debridement, and suturing. In cases of acute pulpitis (K04.0) and acute apical periodontitis (K04.4), the emergency treatments performed consisted of pulp drainage and pulpectomy procedures; hence, these treatment measures, pulpal cavity disinfection, and pain relief appeared to have increased during the SARS-COV-2 pandemic. Acute pericoronitis (K05.2) mainly occurs in the 19–45-year age group [25]. During an infection, tooth extraction might lead to the spread of infection, and at the same time, it might increase the risk of infection with SARS-COV-2. Therefore, acute pericoronitis treatment should be a local treatment, supplemented by antibiotics and analgesics. For abscesses, a small incision is needed for draining pus, and in case of space infection, systemic anti-inflammatory treatment might be necessary [26]. The present study showed that trauma accounted for 20.1% during the SARS-COV-2 pandemic, and although it had decreased compared to the time before the pandemic, it was still the main dental emergency among minors during the pandemic. Previous studies have shown that the 0–18-year age group is more likely to sustain traumas [15, 27]. Parents with children in emergency rooms are more anxious because of the pandemic [28]. Therefore, dentists in charge of dental emergency rooms should be familiar with dental trauma in the primary and mixed dentition. In particular, behavior control is difficult in children with trauma, and since the guardians can be sensitive and anxious, dentists should be well-trained for the behavioral control of the patient and guardian, their prognosis, and follow-up measures. If necessary, knowledge of drugs and emergency treatment is also required, as pediatric patients might need to be sedated through medications [19, 29]. During the SARS-COV-2 pandemic, the emergency treatment principle of trauma was to check the patient’s overall condition, whether there was any injury to the brain, chest, abdomen, and important organs, with the dental emergency doctor assessing the dental status in preparation for surgical suturing of the injury or wound after excluding any life-threatening conditions [16].

The literature shows the inappropriate use of antibiotics, especially in dental emergency centers. Tulip et al. reported that 50% of patients were treated with antibiotics alone without any local treatment [30]. In our survey, before the SARS-COV-2 pandemic, 17.7% of patients received a prescription for antibiotics and analgesics. During the pandemic, this percentage reached 39.2%. A brief review of the data on antibiotics shows that this ratio will be even lower. Therefore, this study’s data can show that the use of antibiotics was relatively standardized, which might be attributed to the impact of the epidemic. When patients are relatively optimistic, the use of antibiotics and analgesics increases, shortening the time doctors contact patients and reducing the risk of infection. However, education about the appropriate use of antibiotics and an update of dentists’ knowledge is mandatory to avoid unnecessary prescription of antibiotics [30, 31].

During the SARS-COV-2 pandemic, dental emergency services experienced an increase in their workload, reflecting the changes in the dental emergency spectrum as the pandemic progressed. The rational distribution of dental practitioners could compensate for the shortcomings mentioned above in the dental emergency departments and help mobilize more emergency resources of dental assistants and hygienists [12, 15]. It would also be necessary to train stomatologists to have sound professional knowledge and surgical skills in dental medicine to meet the technical requirements of potential dental emergencies and ensure that all the conditions can be dealt with quickly and effectively within the emergency department [2, 11]. Simultaneously, the dental emergency department should reasonably define and adhere to pandemic preventive measures and sufficiently stock common anti-inflammatory and analgesic drugs to meet the patients’ medical needs. The results might be an improvement in the operation efficiency of emergency treatments in terms of professional technology, personnel, and materials available [31].

## Conclusion

SARS-COV-2 pandemic led to changes in the characteristics of dental emergency patients. Trauma, acute pulpitis, and acute periodontitis were the leading causes of patients visiting the dental emergency. The dental emergency department should optimize the treatment procedures, optimize the staffing, and reasonably allocate materials according to the changes to improve the on-site treatment capacity and provide adequate dental emergency care.

## Data Availability

The datasets used and/or analyzed during the current study are available from the corresponding author on reasonable request.

## Abbreviations

SARS-CoV-2: Severe acute respiratory syndrome CoV-2
ICD-10: International Classification of Diseases-10
